# Pulse Field Ablation: An Update on Energy-Specific Adverse Events

**DOI:** 10.31083/RCM44442

**Published:** 2025-10-23

**Authors:** Minghao Zhou, Hongliang Yang, Huan Sun, Ming Yu, Daoyuan Si, Yuquan He

**Affiliations:** ^1^Department of Cardiology, China-Japan Union Hospital of Jilin University, Jilin Provincial Molecular Biology Research Center for Precision Medicine of Major Cardiovascular Disease, 130033 Changchun, Jilin, China

**Keywords:** atrial fibrillation, pulsed field ablation, energy-specific adverse event, vagal ganglionated plexus, coronary artery spasm, hemolysis

## Abstract

Pulsed field ablation (PFA) represents a paradigm shift in cardiac ablation technology. Indeed, PFA, a non-thermal technique, achieves homogeneous tissue effects by delivering ultrashort, high-frequency electric pulses. Moreover, PFA has emerged as a prominent ablation strategy in electrophysiology laboratories worldwide. While current clinical evidence demonstrated promising outcomes and expanded applications for existing PFA platforms, energy-specific difficulties complicated the definition of safety boundaries, particularly in patients with clinical complications. Furthermore, substantial heterogeneity among commercial PFA systems impeded procedural standardization. Therefore, this review aimed to synthesize contemporary perspectives on mitigating these energy-specific adverse events, with emphasis on modifiable perioperative factors. The evidence of safety across commercially available PFA systems with divergent catheter design philosophies was concurrently evaluated to examine how design strategies influence procedural safety profiles.

## 1. Introduction

Pulsed field ablation (PFA) technique has emerged as an advanced therapeutic approach for arrhythmias, representing a significant improvement in cardiac ablation technique [1, 2]. It has attracted much attention as a new form of ablation energy and for its preferential myocardial selectivity and effectiveness in forming homogeneous lesions. However, registry data based on the real-world applications for atrial fibrillation (AF) treatment indicated that 0.2% of patients experience energy-specific adverse events (AEs) [3], which represent a critical aspect of PFA safety assessment. Recent statistical analyses of data from Farapulse (Boston Scientific) and Pulseselect (Medtronic) devices revealed that 21% (13/61) of patient complications associated with the Farapulse system are attributable to energy-specific AEs [4]. These complications have caused anxiety among PFA operators and have raised concerns regarding the safety associated with different ablation modalities. However, there is no standardized perioperative management strategy to avoid the potential risk of energy-specific AEs due to the strong heterogeneity of contemporary PFA ablation systems in catheter design and ablation parameter settings. Thus, there is an urgent need for a generalized perioperative intervention plan to avoid the occurrence of unpredictable perioperative AEs due to the increasing frequency of PFA application and the continuous expansion of its indications. Therefore, the standardized PFA ablation procedure should advance by summarizing the existing clinical/device factors associated with energy-specific AEs.

## 2. The Classification of Energy-Specific Complications

**Fig. 1.**
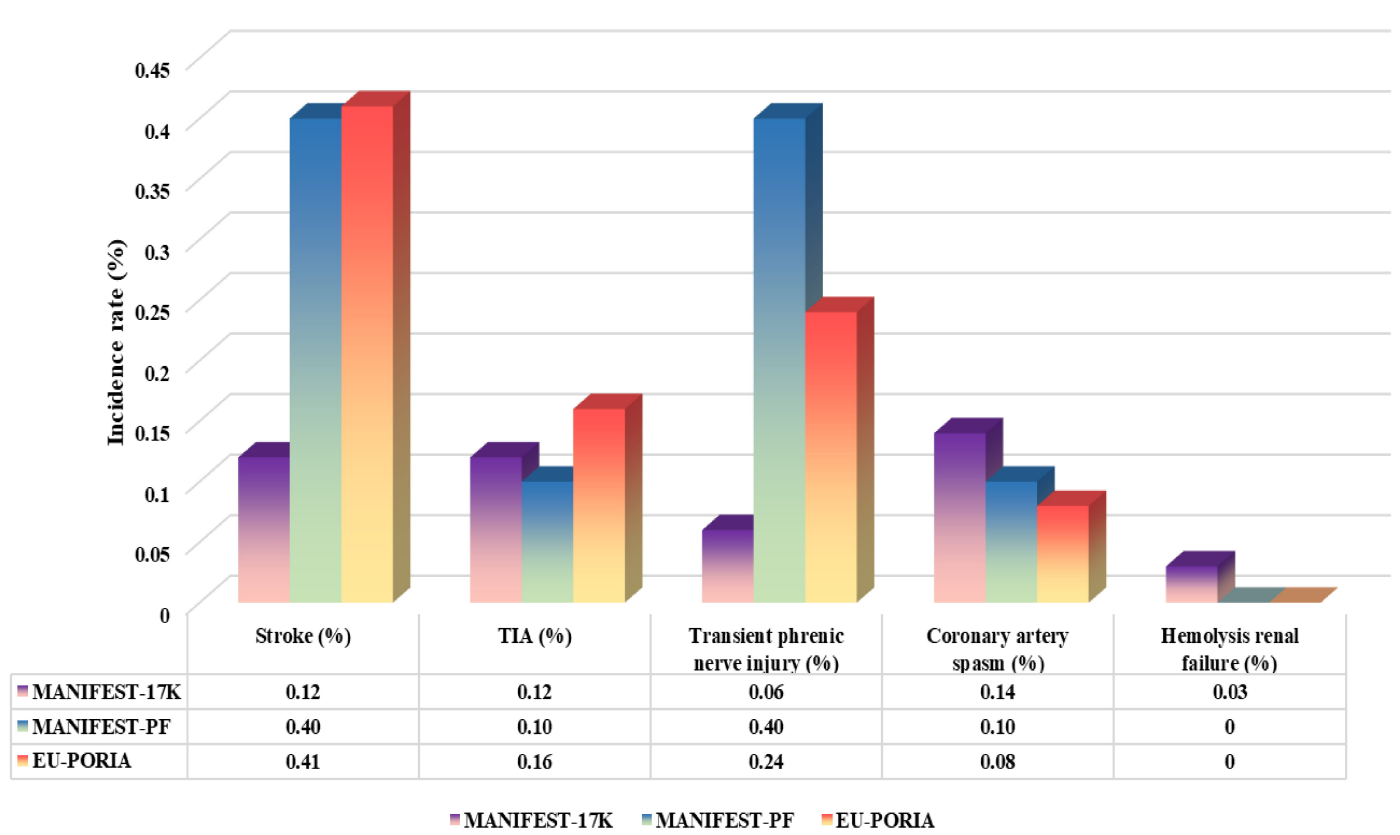
**The incidence of energy specific adverse events of the Farapulse system based on contemporary registration**.

Available meta-analyses indicate that PFA exhibited a superior safety profile when compared to thermal ablation modalities (e.g., radiofrequency ablation [RFA] and cryoballoon ablation [CBA]), particularly regarding serious complications (Table 1) [5, 6, 7, 8, 9, 10, 11, 12, 13, 14]. Nevertheless, PFA is associated with distinct energy-specific AEs, including nerve dysfunction, coronary artery spasm, intravascular hemolysis, and hemolysis-induced acute kidney injury (AKI) [3]. Analysis of large PFA registries (primarily using the Farapulse system) quantified the percentage of the most relevant AEs, as illustrated in Fig. 1 [3, 15, 16]. Specifically, the Multi-National Survey on the Safety of the Post-Approval Clinical Use of Pulsed Field Ablation in 17,000+ Patients (MANIFEST-17K) established definitions for energy-specific AEs identifying risks including transient neurological disturbance (e.g., phrenic nerve palsy and excessive vagal response), vasospasm, and hemolysis-induced renal failure. The analysis of the Food and Drug Administration (FDA) Manufacturer and User Facility Device Experience (MAUDE) database (encompassing 1237 PFA and RFA reports) further delineated energy-specific AEs, thus supporting the above analysis. According to these results, the most frequently reported PFA AEs are pericardial effusion, vasovagal reaction, and hemolysis. This profile is in contrast with that of RFA, where pericardial effusion, ischemic stroke, and esophageal injury are the predominant ones. Vagal reactions (14.1% *vs.* 0%), coronary spasm (5.8% *vs.* 0.6%), and hemolysis (9.0% *vs.* 0%) show significantly higher incidence rates with PFA compared to RFA [17].

**Table 1.** **The pulse field ablation safety analysis based on meta-analysis**.

Author	Included studies	Included patients	Intervention measure	Safety evaluation
Aldaas OM *et al*. [5]	6	1012	Intervention group: PFA (FARAPULSE only)	No statistically significant disparities in periprocedural complications were manifested across comparative cohorts. Notwithstanding this equivalence, complications pathognomonic to PFA, particularly coronary vasospasm, remained insufficiently characterized in the extant literature due to methodological limitations in surveillance protocols and outcome reporting frameworks within the analyzed studies
			Control group: any ablation technique (either RFA or CBA)	
Zhang H *et al*. [6]	15	1880	Intervention group: PFA (FARAPULSE only)	Comparative analysis demonstrated a significantly reduced incidence of transient phrenic nerve palsy in the PFA cohort versus CBA controls (*p * < 0.01), with complete esophageal preservation observed in the PFA group. Notably, pericardial tamponade incidence exhibited modest elevation in PFA recipients (10/775, 1.29%), a phenomenon mechanistically linked to the inherent rigidity of the specialized guidewire integral to PFA catheter deployment
			Control group: CBA only	
Iqbal M *et al*. [7]	20	3857	Intervention group: PFA (FARAPULSE only)	The composite complication rate demonstrated in our meta-analysis exhibited concordance with the recently published ADVENT trial. Comparative analysis revealed numerically reduced frequencies of vascular access complications (0.60% *vs.* 1.31%) and pericardial effusion/tamponade (0.60% *vs.* 0.78%) following PFA. The incidence of cerebrovascular events (stroke/transient ischemic attack: 0.40% *vs.* 0.17%) was observed to be elevated in the PFA cohort compared with conventional ablation approaches
			Control group: any ablation technique (either RFA or CBA)	
Qamar U *et al*. [8]	26	2561	PFA only	Among 2451 patients undergoing PFA, 91 manifested postprocedural complications, yielding an aggregate event rate of 2.8%. Vascular access complications and cardiac tamponade (each 0.60%) constituted the predominant adverse events, succeeded in frequency by transient ischemic attacks (0.40%), cerebrovascular accidents (0.30%), and phrenic nerve injury/palsy (0.30%)
de Campos MCAV *et al*. [9]	18	4998	Intervention group: PFA (Catheter undefined)	Reduced incidence of esophageal injury and increased occurrence of cardiac tamponade were associated with PFA, a phenomenon potentially attributable to the enhanced structural rigidity of the guidewire utilized in PFA catheter deployment. While no instances of hemolysis-associated acute kidney injury were documented, the potential clinical ramifications of this observation merit ongoing scientific scrutiny
			Control group: any ablation technique (either RFA or CBA)	
Rudolph I *et al*. [10]	11	3805	Intervention group: PFA (Catheter undefined)	Quantitative analysis demonstrated statistically significant attenuation of perioperative complications in the PFA cohort compared with CBA recipients. The PFA group exhibited a marked reduction in phrenic nerve injury incidence and complete absence of esophageal thermal lesions, contrasting with a paradoxically elevated risk of pericardial tamponade. No intergroup disparities were observed in vascular access complications or cerebrovascular events encompassing transient ischemic attacks and stroke
			Control group: CBA only	
Amin AM *et al*. [11]	17	2255	Intervention group: PFA (FARAPULSE/HexaPulse)	PFA demonstrated significant reductions in postprocedural heart rate variability (*p * < 0.01), phrenic nerve palsy incidence (*p* = 0.05), and esophageal lesion formation (*p* = 0.02) compared with thermal ablation modalities. Paradoxically, PFA exhibited an elevated pericardial tamponade incidence. No intergroup differences reached statistical significance in composite complication rates, cerebrovascular (stroke/TIA)/thromboembolic events, or all-cause mortality
			Control group: any ablation technique (either RFA or CBA)	
Waseem MH *et al*. [12]	7	1538	Intervention group: PFA (Catheter undefined)	Comparative analysis revealed no significant intergroup disparities in composite complication rates between PFA and vHPSD ablation cohorts (*p* = 0.88). Stratified subgroup analyses further demonstrated equivalent risk profiles for pericardial tamponade (*p* = 0.75) and cerebrovascular events (*p* = 0.84)
			Control group: any ablation technique (vHPSD)	
Xue J *et al*. [13]	6	1382	Intervention group: PFA (FARAPULSE only)	Comparative analysis demonstrated no statistically significant interprocedural disparities in perioperative complication profiles between PFA and vHPSD ablation cohorts (*p* = 0.91). Notably, complete procedural preservation of phrenic nerve integrity and atrioesophageal continuity was observed across both groups. The PFA cohort exhibited one procedure-related fatal cerebrovascular accident, while the vHPSD group manifested a single incident of pulmonary venous stenosis
			Control group: any ablation technique (vHPSD)	
Kaddoura R *et al*. [14]	30	7167	Intervention group: PFA (FARAPULSE/Disposable PFA8D18L catheter)	Comparative analysis revealed PFA demonstrated statistically significant superiority in reducing composite postoperative complication rates compared with CBA, whereas no statistically significant disparity was observed between PFA and RFA. Owing to the infrequency of acute complications across cohorts, the investigation did not conduct stratified analyses of immediate procedural sequelae. Notwithstanding this limitation, emerging safety considerations specific to PFA, particularly pericardial tamponade and coronary vasospasm, warrant heightened clinical vigilance
			Control group: any ablation technique (either RFA or CBA)	

ADVENT, atrial fibrillation study evaluating new technology; TIA; transient ischemic attack; PFA, pulsed field ablation; RFA, radiofrequency ablation; CBA, cryoballoon ablation; vHPSD, very High-Power Short-Duration radiofrequency ablation.

Debates regarding the cerebrovascular risks of PFA persist. Despite most studies report comparable rates of stroke/transient ischemic attack (TIA) to thermal ablation, concerns remain on the potential undefined cerebral injury risks [7, 14]. *In vitro* studies revealed that gas microbubbles (20–200 µm in diameter) formed selectively in the anode region of PFA devices, contribute to acute ischemic events, and significant differences in bubble formation are observed among current commercial systems [18, 19]. The real-time carotid echocardiography that detects the formation of microbubbles revealed that Varipulse generates substantially more microembolic signals (MESs), silent cerebral events, and silent cerebral lesions than other PFA platforms. However, further investigation is needed to confirm that MESs induce impaired neurological or cognitive function. Forleo GB *et al*. [20] noted that microemboli from catheter ablation approach levels seen in major cardiac surgery, which may drive subtle neuropsychological changes, including higher rates of cognitive impairment. However, as most ischemic signals resolve within 90 days without chronic glial scarring, microbubble formation alone cannot fully define the neurosafety profile of PFA. RFA emboli typically occur during sheath/catheter exchange, while CBA emboli occur during catheter insertion or balloon inflation. However, sheath replacement in PFA may introduce abundant gas emboli that likely result in extensive MES formation, suggesting this step as a potential target for mitigating post-ablation neural injury. Since the specific mechanisms underlying potential PFA-related cerebral injury remain incompletely elucidated, the discussion on energy-specific AEs is focused on those with established associations: vagal responses, coronary spasm, hemolysis, and hemolysis-induced AKI.

## 3. Available Factors for Energy-Specific Aes Perioperative Intervention

### 3.1 Intraoperative Vagal Response

#### 3.1.1 Competitive Muscarinic Antagonist

**Fig. 2.**
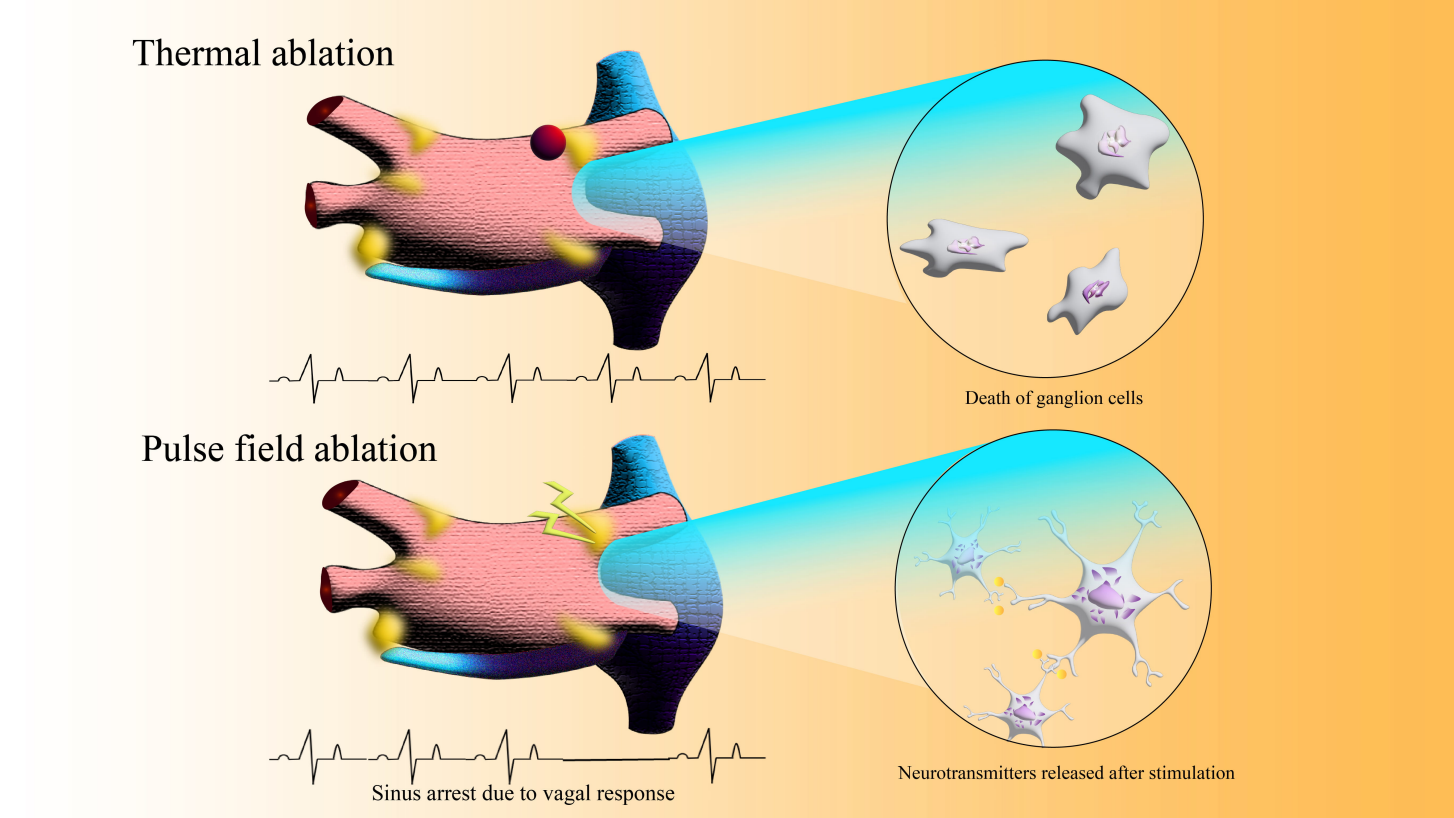
**Electrocardiogram changes and local vagal response in patients after thermal ablation or after PFA during reablation**.

PFA possesses a unique neurotropic selectivity that preserves vagal ganglionic cell viability while eliciting transient autonomic stimulation [21], evidenced by the absence of significant heart rate increase [22] and elevated post-procedural S100 protein levels [23], the former being a biomarker associated with denervation, as shown in Fig. 2. Experience based on current CBA application revealed that, preoperative intravenous administration of 1 mg atropine reduces severe vagal reactions from 92.3% (12/13) to 33.3% (4/12; *p* < 0.01) [24]. Current PFA protocols do not require their prophylactic administration, while competitive muscarinic antagonists such as atropine and glycopyrrolate are frequently used to mitigate excessive vagal response. This situation requires critical re-evaluation due to the procedural demands of PFA, particularly the repeated pulsed field applications required to achieve sufficient lesion formation in the targeted anatomical regions [25, 26], substantially increasing the risk of vagal activation through electrical stimulation of the cardiac plexuses. Future procedural guidelines should explicitly address the management of intraoperative vagal response by incorporating standardized strategies such as preprocedural or intraprocedural application of competitive muscarinic antagonists and selective use of temporary ventricular pacing leads.

#### 3.1.2 Initial Ablation Site

**Fig. 3.**
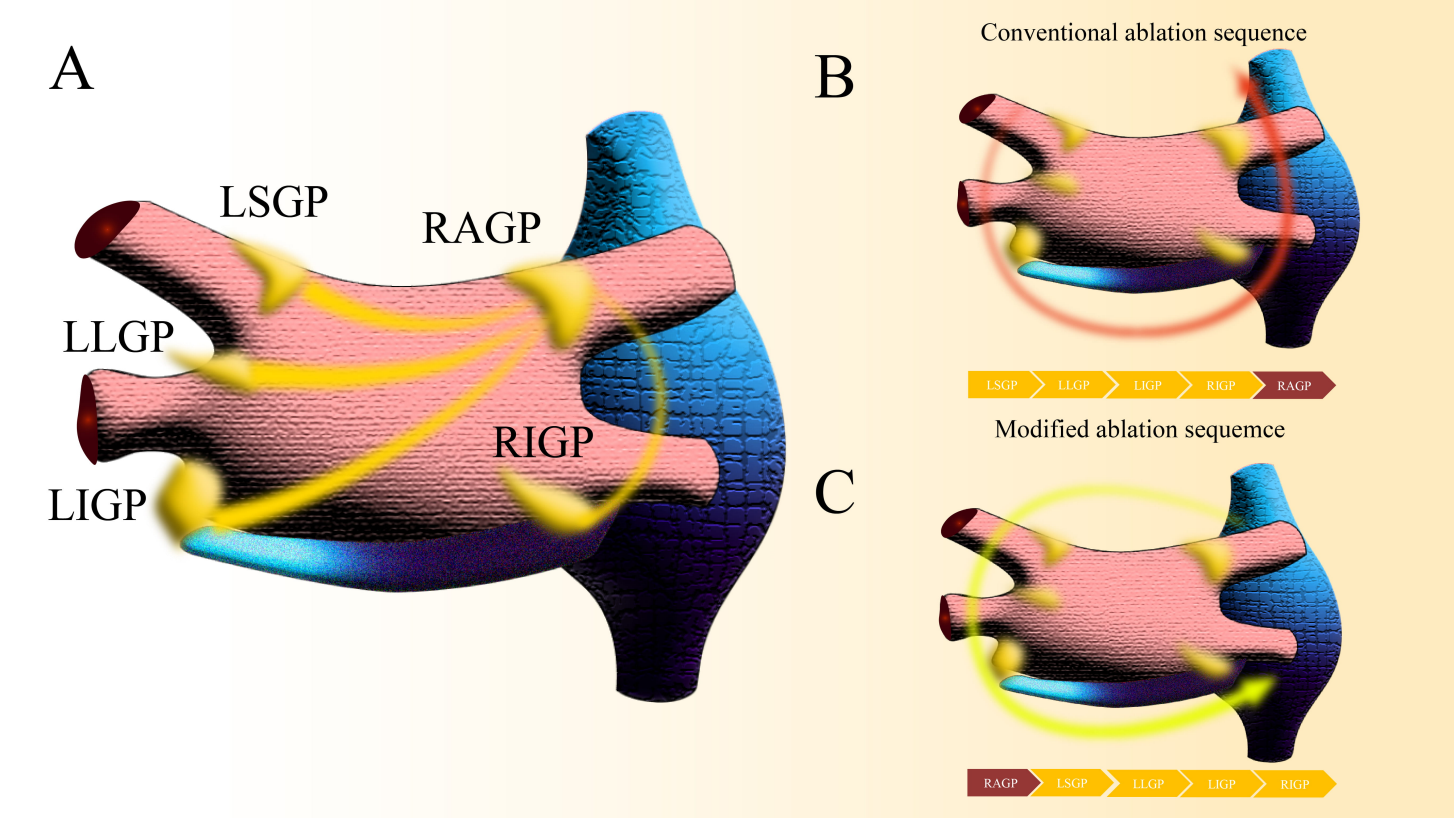
**Modified ablation based on right superior vagal plexus modulation**. (A) Gross distribution of the cardiac vagal plexus. (B) Conventional ablation sequence of PVI. (C) Modified ablation sequence of PVI. PVI, Pulmonary vein isolation; LIGP, Left Inferior Ganglionated Plex; LLGP, Left Lateral Ganglionated Plex; LSGP, Left Superior Ganglionated Plex; RAGP, Right Anterior Ganglionated Plex; RIGP, Right Inferior Ganglionated Plex.

The vagal ganglia localized in the subepicardial fat pads adjacent to the myocardial layer constitute critical mediators of neurocardiac regulation and represent strategic therapeutic targets for autonomic reflex modulation during cardiac ablation. These neural aggregates function as interconnected networks in the cardiac autonomic architecture (Fig. 3A). Hu F *et al*. [27]. confirmed that prioritizing intervention and regulation of the right anterior ganglionic plexus (RAGP) during PFA (Fig. 3B,C) significantly reduces the incidence of intraoperative vagus nerve involvement (2.5% *vs.* 62.5%). This RAGP-centric approach has equivalent efficacy across RFA modalities. Current prospective PFA data reveal a remarkable disparity in autonomic responses: an left superior pulmonary vein (LSPV)-first strategy elicit vagal reactions in 78% of cases versus 13% with right superior pulmonary vein (RSPV) prioritization. Furthermore, the RSPV-focused protocol significantly reduces temporary pacing requirements (35% *vs.* 8%; *p* < 0.01) [28].

### 3.2 Coronary Spasm

#### 3.2.1 Prophylactic Nitroglycerin Administration

**Fig. 4.**
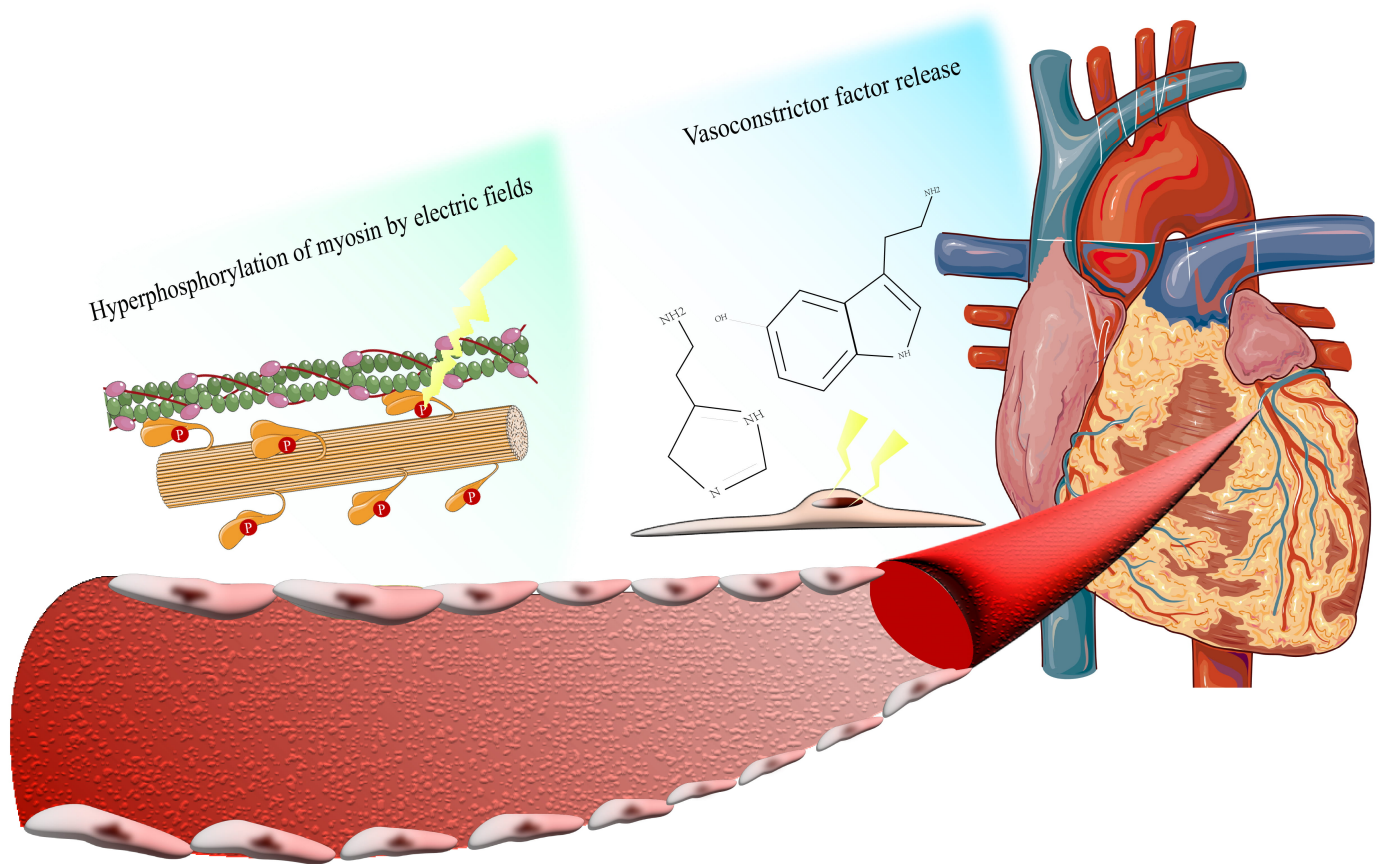
**Intraoperative coronary spasm mediated by myosin hyperphosphorylation and local vasoconstrictor release**.

The coronary spasm induced by PFA is primarily due to direct stimulation of the vascular smooth muscle by pulsed electric fields (PEF) and the cascade of local inflammatory responses [29, 30], as shown in Fig. 4. The reported incidence of such acute vascular reactions during PFA is from 0.08% to 0.14% [3, 15, 16, 31]. Intravenous nitroglycerin is frequently used as a prophylactic measure during the ablation of the anatomic site adjacent to the coronary artery [32]. However, the administration of this pharmacological treatment does not universally preclude the intracoronary administration to alleviate coronary spasm in all patients. Even with the prophylactic use of nitroglycerin, a subset of patient still experiences delayed and, clinically significant coronary spasm. A pertinent case report documented the occurrence of clinically apparent vasospasm induced by a pentaspline PFA catheter during cavotricuspid isthmus (CTI) ablation, despite the prophylactic administration of high-dose intravenous nitroglycerin prior to PFA. This episode was characterized by a significant vasospasm accompanied by a ST-segment elevation in leads II, III, and aVF on the electrocardiogram. The clinical scenario was further complicated by the onset of hemodynamically unstable nonsustained ventricular tachycardia, culminating in cardiac arrest in need of temporary pacing and an additional 4 mg nitroglycerin [33]. Our hypothesis was that the transient cellular electroshock effect induced by PEF further prolonged the metabolic clearance time of the aforementioned hyperphosphorylated myosin light chain, while reducing vascular smooth muscle sensitivity to nitric oxide, consequently exacerbating vasoconstrictor responses and increasing nitric oxide dosage requirements. As regards the increase of the efficacy of nitrate administration, Malyshev Y *et al*. [34] propose two innovative nitroglycerin delivery protocols: (1) multiple bolus injections (2–3 mg every 2 minutes) into the right atrium, and (2) a single bolus injection (3 mg) into the right atrium combined with continuous peripheral intravenous infusion (1 mg/min). These strategies show a significant effect in preventing severe spasm, although the optimal dosing regimen remains to be established due to the limited sample size in their study.

#### 3.2.2 Anatomical Sites

**Fig. 5.**
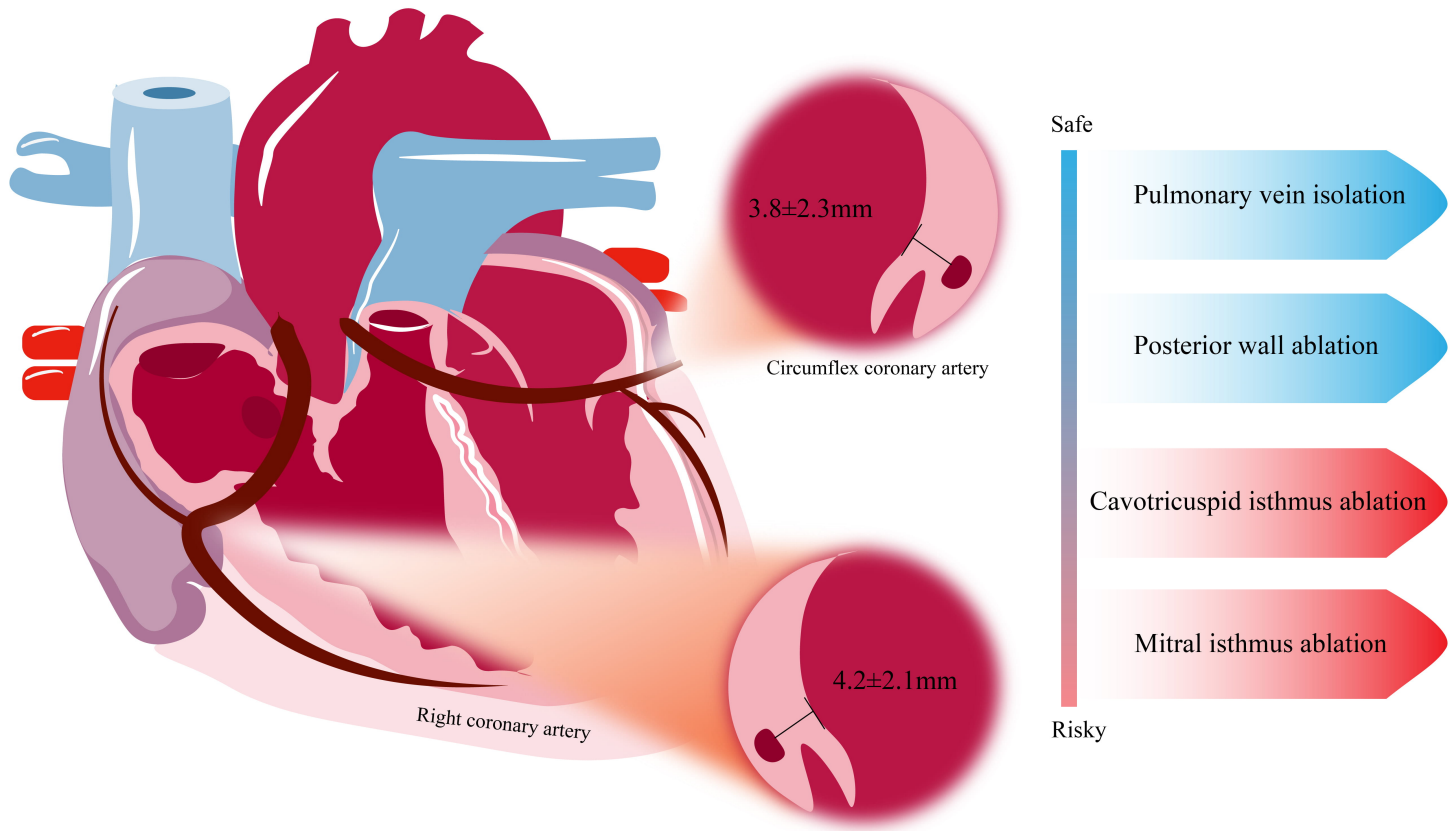
**The anatomical basis of coronary artery spasm and the response of different ablation sites to pulsed electric fields**.

PFA-induced coronary spasm shows distinct clinical characteristics, including acute onset patterns and specific anatomical predilections [32, 35, 36, 37, 38, 39, 40]. The mean distance between the endocardium and the right coronary artery at the CTI is 4.2 ± 2.1 mm, while the minimum distance at the mitral isthmus (MI) region is 3.8 ± 2.3 mm (Fig. 5) [41]. However, the ideal tissue proximity is identified as a significant factor influencing the formation of PFA-induced electrical isolation [42]. Clinical experience from pentaspline PFA catheter confirms that coronary spasm frequently occurs during the linear ablation of the CTI and superior MI [32, 43]. Most such cases involve subclinical vasospasm induced by localized electric field stimulation. However, optical coherence tomography observations by Tam MTK *et al*. [44] reveal significant vascular remodeling three months post-PFA, as the median vessel wall area increases by 17.1% and the lumen area decreases by 10.1%. Potential hemodynamic consequences need attention due to the established correlation between atherosclerosis severity and coronary spasm [45], particularly regarding PFA exacerbating flow impairment in patients with pre-existing coronary abnormalities such as atherosclerotic plaques or myocardial bridges.

### 3.3 Intravascular Hemolysis and Subsequent Acute Renal Injury

**Fig. 6.**
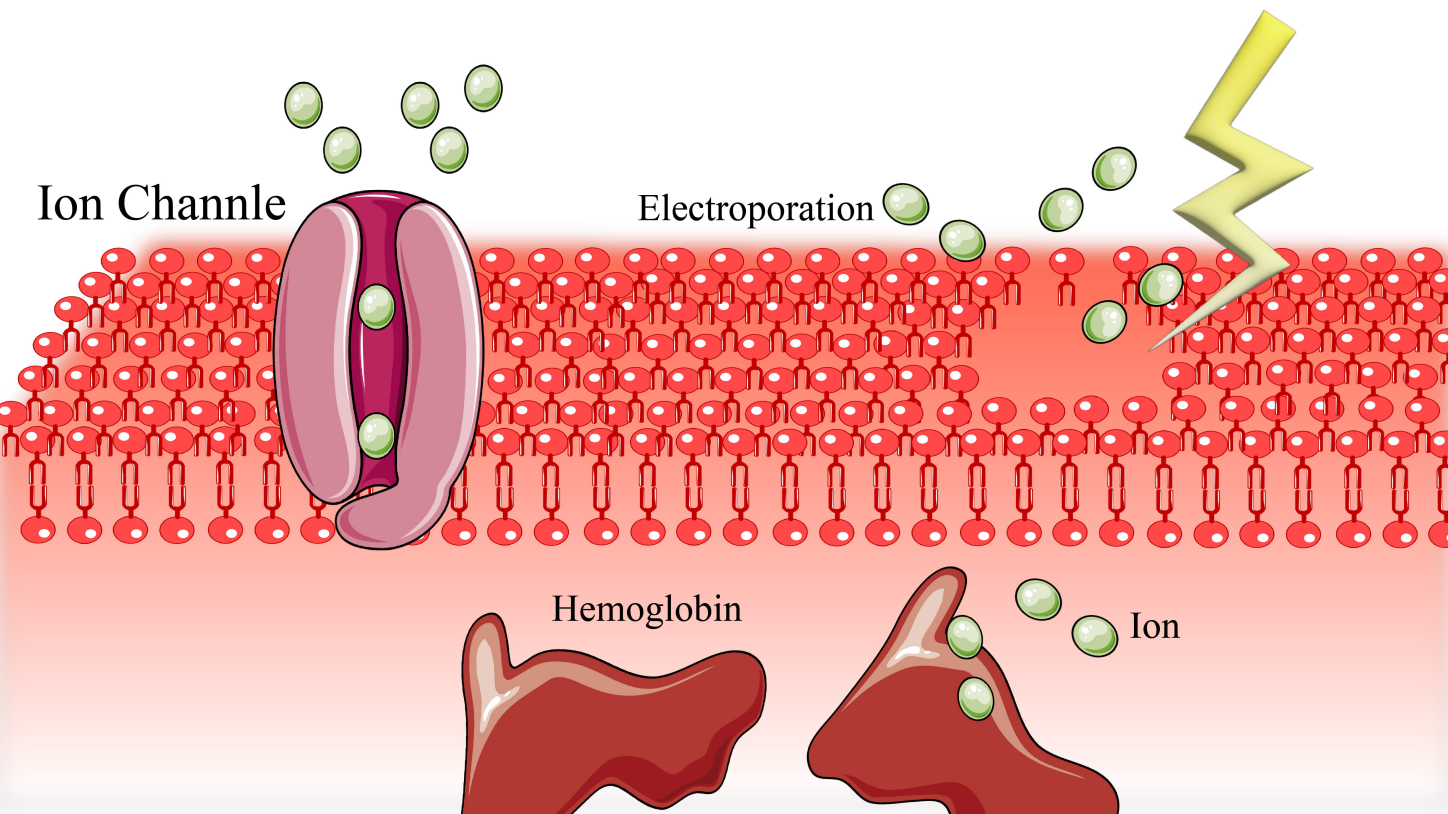
**Focal ion influx mediated by electroporation results in colloid osmotic**.

The exposure of erythrocytes to high-voltage pulses (several kV/cm) generates a transmembrane potential, leading to pore formation in the cell membrane at critical thresholds (Fig. 6). This membrane permeabilization results in colloid osmotic hemolysis, characterized by erythrocyte swelling and rupture due to hemoglobin-induced osmotic pressure [46, 47]. Severe hemolysis can precipitate AKI through heme-mediated proximal tubule epithelial damage and intratubular protein condensation, manifesting as oliguria and increased creatinine levels [48]. Current studies related to PFA hemolysis and AKI are summarized in Table 2 (Ref. [49, 50, 51, 52, 53, 54, 55, 56, 57, 58, 59]), which helps to determine reasonable perioperative management strategies.

**Table 2.** **Summary of related studies on PFA-induced hemolysis and AKI**.

Author	Year	Sample	Catheter	Influencing factors	Results
De Smet MAJ *et al*. [49]	2024	198 consecutive patients (65% Pulmonary vein isolation-only, 35% PVI with additional lesion sets)	Varipulse variable loop PFA catheter (Biosense Webster)	Type of catheter	1. Peri-procedural hemolysis occurs in a significant proportion of patients following PFA
			Farawave pentaspline PFA catheter (Boston Scientific)	Energy (RF *vs.* PF)	2. PVI associated with additional lesion sets can increase the risk of hemolysis and AKI
			ThermoCool SmartTouch (Biosense Webster)	Patient baseline characteristics	3. Hemolysis occurs regardless of the type of PFA catheter used
					4. AKI patients are generally older, more likely received PVI-plus ablation, and have CKD, heart failure, diabetes mellitus, and arterial hypertension
					5. Prognosis of AKI induced by PFA is good, except for patients with advanced CKD
Popa MA *et al*. [50]	2024	215 consecutive patients (49.8% Paroxysmal atrial fibrillation, 50.2% Persistent atrial fibrillation)	ThermoCool SmartTouch SF (Biosense Webste)	The total number of PFA applications	1. Intravascular hemolysis is a frequent finding after PFA for AF and increases with the number of PFA deliveries (>54 PFA deliveries)
			QDOT MICRO (Biosense Webste)	Energy (RF *vs.* PF)	2. Patients with baseline GFR <50 mL/min treated with PFA experience a transient deterioration of renal function and may therefore be at risk of developing AKI
			Farawave pentaspline PFA catheter (Boston Scientific)	Patient baseline characteristics	
Venier S *et al*. [51]	2023	68 consecutive patients (35% Paroxysmal atrial fibrillation, 29% Persistent atrial fibrillation, 35% Long-standing persistent AF)	Farawave pentaspline PFA catheter (Boston Scientific)	The total number of PFA applications	1. There is an inverse correlation between plasma haptoglobin levels and the total number of PFA applications
					2. More than 70 PFA applications can predict hemolysis
					3. Acute kidney injury only in cases where the total number of applications has exceeded 100
Osmancik P *et al*. [52]	2024	70 consecutive patients (51.4% Paroxysmal atrial fibrillation, 48.6% Persistent atrial fibrillation)	Farawave pentaspline PFA catheter (Boston Scientific)	The total number of PFA applications	1. The occurrence of hemolysis is more common during PFA than during RFA in AF ablation
			ThermoCool SmartTouch or QDot (Biosense Webster)	Energy (RF *vs.* PF)	2. The extent of hemolysis depends on the number of PF applications
					3. Additional ablation leads to significantly higher of peak concentration of RBCµ at the end of procedure
Stojadinović P *et al*. [53]	2024	60 consecutive patients (51.7% Paroxysmal atrial fibrillation, 48.3% Persistent atrial fibrillation)	Farawave pentaspline PFA catheter (Boston Scientific)	The total number of PFA applications	1. Patients with more than 74 PEF applications from the pentaspline ablation catheter are at risk of having major hemolysis
Jordan F *et al*. [54]	2024	2570 retrospective patients cohort (66.4% RFA, 21.7% CBA, 11.9% PFA)	ThermoCool or SmartTouch SF (Biosense Webste)	Energy (RF *vs.* CB *vs.* PF)	1. The incidence of AKI for PFA (1.0%) is very low in this large cohort of 2570 patients
			Arctic Front (Medtronic) or PolarX (Boston Scientific)		2. AKI is rare when PFA is used in a standardized fashion with no extensive high number of applications
			Farawave pentaspline PFA catheter (Boston Scientific)		
Mohanty S *et al*. [55]	2024	103 consecutive patients (72% Paroxysmal atrial fibrillation, 28% Persistent atrial fibrillation)	Farawave pentaspline PFA catheter (Boston Scientific)	The total number of PFA applications	1. The number of PF applications and postablation hydration are independent predictors of renal insult
				Postablation hydration	2. Using adequate fluid infusion immediately after the procedure can prevent AKI
Nies M *et al*. [56]	2024	76 blood samples from 4 swine	Farawave pentaspline PFA catheter (Boston Scientific)	The total number of PFA applications	1. Hemolysis related to PFA occurs in a dose-dependent manner
				Catheter-Tissue contact	2. PFA with no-contact application induces more pronounced hemolysis
Mattison L *et al*. [57]	2024	6 swine	PulseSelect PFA system (Medtronic)	The total number of PFA applications	1. Increased number of applications causes more hemolysis
				Catheter-Tissue contact	2. Linear fit shows an increase in PFH of 0.0003 g/dL per application when in contact and 0.00056 g/dL per application when not in contact
Fiserova I *et al*. [58]	2025	Blood samples from healthy volunteers Mouse HL-1 cardiomyocyte cell lines	Tonapulse electrical pulse generator with an electrode plate (Tonagena, Kladno, Czech Republic)	Electric field intensity	1. Statistically significant hemolysis *in vitro* PFA occurs around 1000 V/cm
					2. Destruction of a cell line of cardiomyocytes started at 750 V/cm, but the highest percentage of cell death (about 75% of exposed cardiomyocytes) occurred at 1500 V/cm
					3. An electric field strength energy of around 1500 V/cm is required for effective induction of cardiomyocyte death, but at electric field strengths greater than 1000 V/cm, PFA causes significant erythrocyte rupture
Yuan J *et al*. [59]	2024	Subjecting fresh heparinized rat blood	Not mentioned	Gd-DTPA application	1. Gd-DTPA concentrations of 100 µM and 1000 µM significantly mitigated hemolysis caused by PFA application
					2. Preadministration of Gd-DTPA effectively reduced erythrocyte destruction and intravascular hemolysis after PFA

RF, radiofrequency; PF, pulsed field; AKI, acute kidney injury; CKD, chronic kidney disease; GFR, glomerular filtration rate; Gd-DTPA, gadolinium-diethylenetriamine pentaacetate.

#### 3.3.1 Patient Condition

The reported incidence of AKI following hemolysis in PFA procedures ranges from 0% to 5.26% among clinical series [49, 50, 51, 52]. A prospective analysis by De Smet MAJ *et al*. [49] identifies AKI-prone patients as older individuals with comorbidities including chronic kidney disease, heart failure, diabetes, and hypertension, typically requiring extended duration of the ablation. Among them, a patient with stage 3 AKI with a baseline glomerular filtration rate of 14 mL/min developed progressive kidney injury (serum creatinine from 3.58 to 3.79 mg/dL) with hyperkalemia after only 32 pentaspline PFA ablations and started to receive renal replacement therapy. Previous studies demonstrated that patients with a baseline glomerular filtration rate <50 mL/min exhibit a statistically significant increase in serum creatinine levels after PFA (Δcrea: 27.0 ± 103.1 *vs.* –0.2 ± 12.1 µmol/L; *p* = 0.010) [50]. This finding suggests that individuals with pre-existing significant renal impairment may represent a high-risk population for PFA-related complications rather than obtaining clinical benefit from the procedure. More importantly, patients with significant preoperative renal dysfunction may experience substantial renal deterioration even with standard PFA protocols.

#### 3.3.2 Application Frequence

**Fig. 7.**
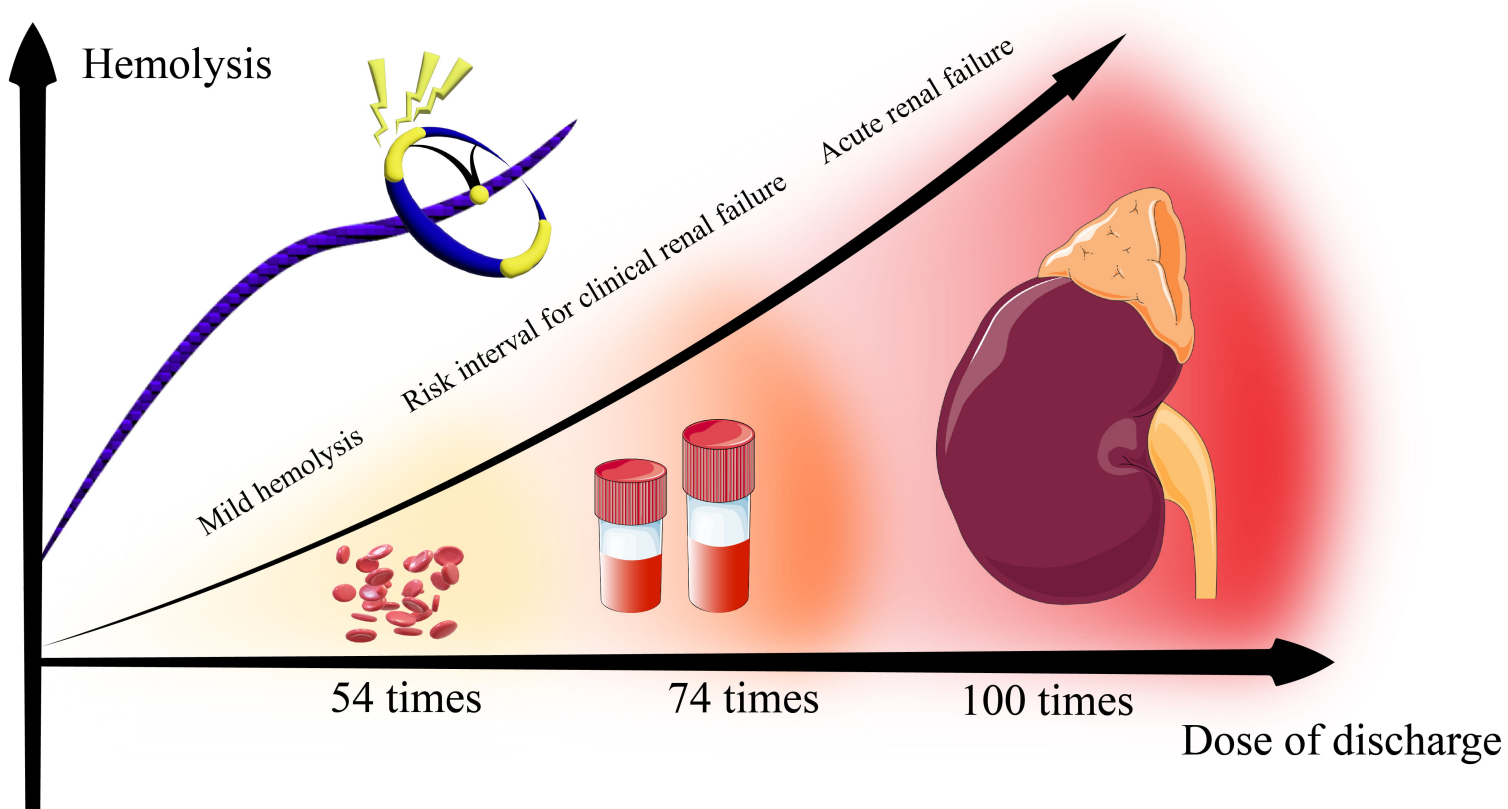
**Effect of intraoperative discharge dose on hemolysis**.

The cumulative number of PFA applications is considered as the most significant intraprocedural determinant of postprocedural hemolysis and renal dysfunction. Venier S *et al*. [51] pioneered the investigation of AKI after PFA, demonstrating a significant inverse correlation between plasma haptoglobin levels and total application count (median [Interquartile Range]: 75 [62–127] *vs.* 62 [54–71] in hemolytic-positive *vs.* negative cohorts, *p* = 0.011). Subsequent validation studies further corroborated these findings, establishing a predictive application threshold range from 54 to 74 discharges for hemolysis risk [50, 51, 53]. Receiver operating characteristic (ROC) curve analysis identifies 70 applications as an optional predictive threshold (area under the curve (AUC): 0.709) with balanced sensitivity and specificity. Procedural safety of Farapulse system can be stratified by discharge frequency (Fig. 7). Hemolysis risk is minimized with less than 54 PFA applications, whereas applications between 54 and 74 markedly increase hemoglobinuria incidence. This dose-dependent effect is particularly significant in patients with persistent AF requiring extensive ablation [49, 52, 54, 60]. Tamirisa KP *et al*. [61] show that application thresholds must be individualized according to the specific PFA system used. Since catheter-induced hemolysis correlates with non-contact electrode exposure in the atrial blood pool, discharge thresholds derived from pentaspline experience cannot be broadly extrapolated to standardize PFA procedures. Although clinical guidelines currently lack specific discharge thresholds for PFA, our recommendations are the following: (1) prioritize focal ablation or single catheters with minimal footprint when anticipating extra ablation; (2) use sensitive biomarkers as red blood cell microparticles to evaluate hemolysis severity. Since red blood cell microparticles are not routinely used in clinical practice, lactate dehydrogenase, haptoglobin, indirect bilirubin and plasma-free hemoglobin may serve as the preferred alternative parameters [61].

#### 3.3.3 Catheter-Tissue Proximity

Nies M *et al*. [56] performed *in vitro* experiments to investigate the relationship between tissue contact and hemolysis using a pentaspline catheter. Analysis of 76 blood samples from four swine indicates that non-contact applications induce significantly greater hemolysis, with free plasma hemoglobin (fHB) levels becoming statistically significant after four applications (0.12 ± 0.03 *vs.* 0.05 ± 0.03 g/dL; *p* = 0.008). This phenomenon was also observed with loop catheters, where both contact and non-contact groups show a linear dose-response relationship between fHB levels and PFA applications. The fHB increase per application is 0.0003 g/dL in contact and 0.00056 g/dL in non-contact conditions [57]. Although previous experience suggested that the damage by PFA is not entirely dependent on contact force, poor adherence to tissue can further predict the degree of intraoperative hemolysis [58].

#### 3.3.4 Preoperative Drug Pretreatment and Postoperative Hydration

The perioperative treatment is considered the basis for avoiding severe renal insufficiency caused by hemolysis. The gadolinium-diethylenetriamine-penta-acetic acid, a clinically approved magnetic resonance imaging (MRI) contrast medium, was introduced for reducing PFA-induced hemolysis through erythrocyte membrane stabilization. Preclinical studies reveal that the above acid modulates erythrocyte membrane stability, improving the resistance to electroporation-mediated membrane disruption during PFA [59]. However, this approach is still in the stage of animal model-based validation due to the lack of reliable evidence. Postoperative hydration is the only treatment established in clinical practice to reduce hemolysis-associated kidney injury. Mohanty S *et al*. [55] demonstrated that, the planned intravenous infusion of 0.9% sodium chloride ≥2 L effectively prevents serum creatinine increase in 75 patients after ablation. Despite this approach probably complicated the postoperative management due to the risk of volume overload and subsequent clinical deterioration, this measure is necessary especially for patients with preoperative renal insufficiency.

## 4. Limitation of Contemporary PFA Ablation Catheters

**Fig. 8.**
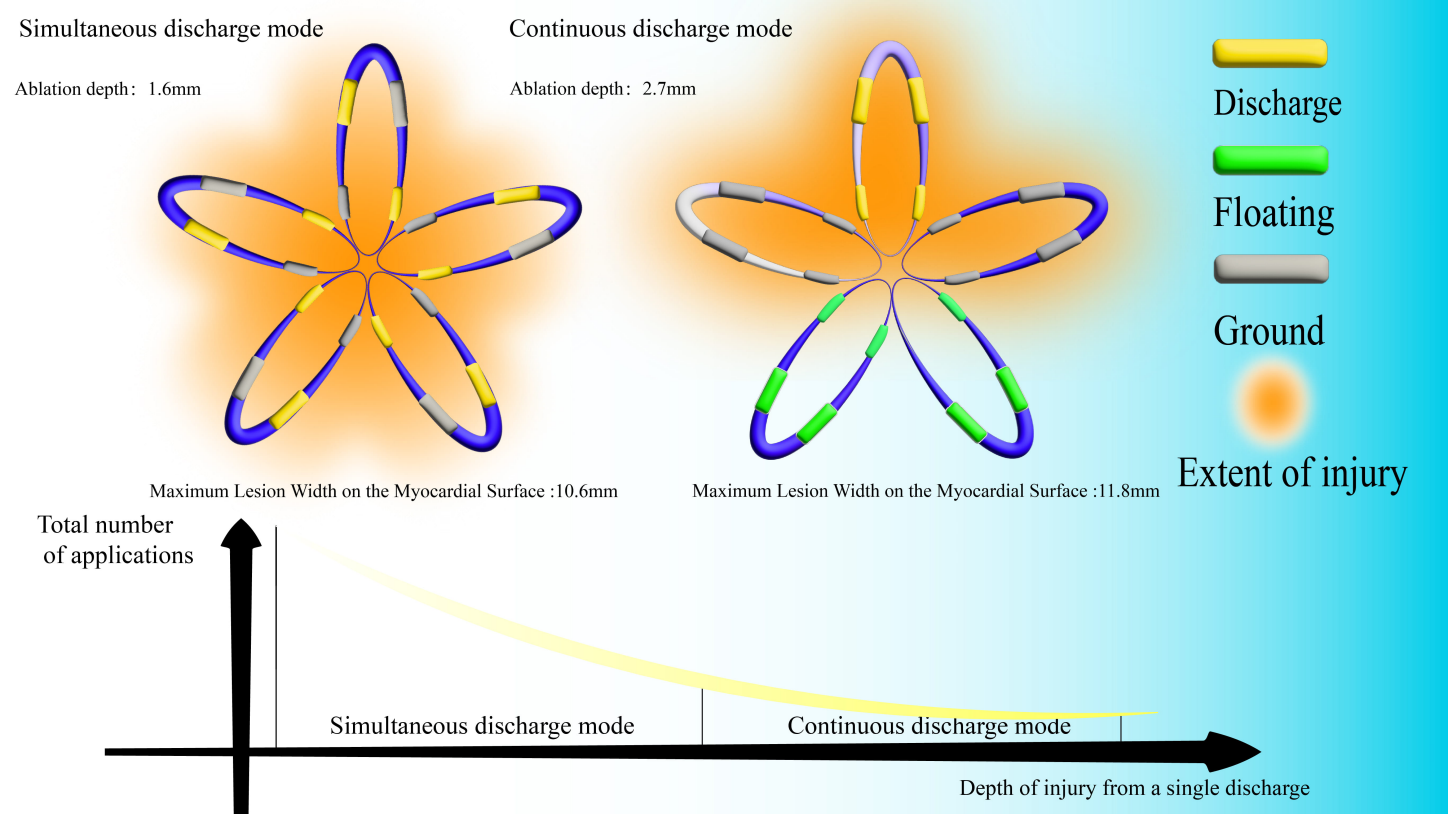
**Comparison of the simultaneous discharge mode and continuous discharge mode**.

Pulse parameters in PFA systems, including voltage, waveform, pulse duration, and frequency, are predefined by manufacturers during device development, resulting in significant inter-device variability. This inherent variability, coupled with the nonlinear relationship between pulse parameters and procedural risks, complicates the establishment of optimal ablation protocols. Contemporary PFA systems predominantly use microsecond-duration pulses, typically applying voltages around 2000 V with bidirectional waveforms. However, the spatial distribution of PEF generated by multielectrode ablation catheters critically depend on the activation modality, which governs whether PEF superpose constructively or cancel each other out. Contemporary pentaspline PFA catheters use two principal energy delivery strategies: simultaneous activation and sequential activation (Fig. 8). Computer simulations showed that the simultaneous activation produces myocardial lesions with a mean depth of 1.6 mm (maximum 3.2 mm). In contrast, sequential activation achieves greater penetration depth (mean 2.7 mm, maximum 5.1 mm) but needs a higher total energy delivery due to the increased application frequency [62]. This escalated energy exposure increases the risk of hemoglobinuria and subsequent renal injury resulting from cumulative erythrocyte destruction.

**Fig. 9.**
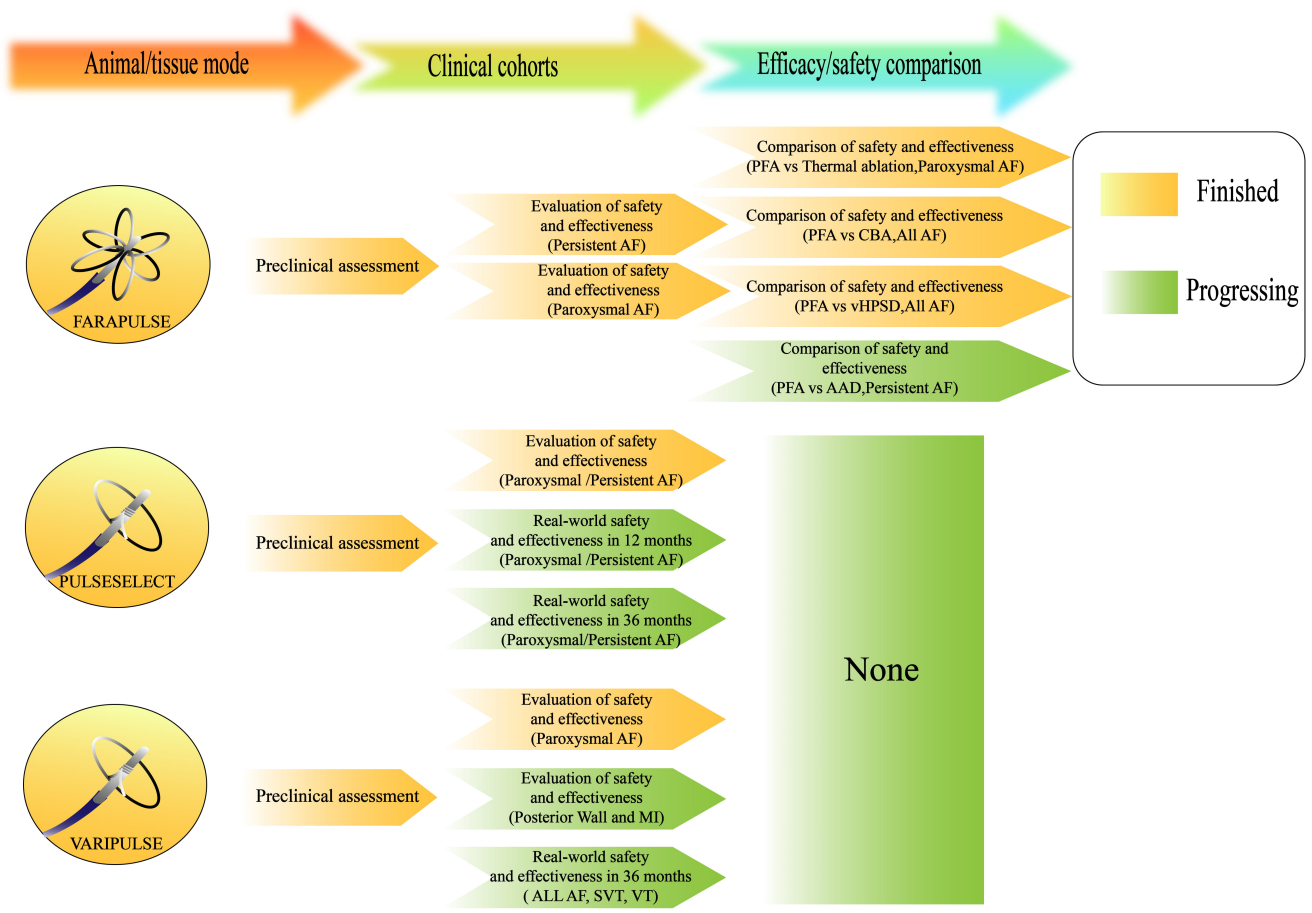
**Research status of the first-generation pulsed field ablation catheters**.

Farapulse (Boston Scientific), Pulseselect (Medtronic), and Varipulse (Biosense Webster) are the PFA systems most widely used worldwide by electrophysiology centers. Among these, Farapulse was subjected to systematic safety and efficacy tests, thus extending its applications beyond pulmonary vein isolation, as validated in the atrial fibrillation study evaluating new technology (ADVENT) trial, to include left atrial posterior wall isolation and CTI linear ablation [63, 64, 65]. As regards further ablation treatments carried out in other anatomical regions, Superior Vena Cava isolation and MI linear ablation have both been proven to be feasible under the PFA procedure [66, 67, 68]. The current clinical research progress on contemporary PFA systems is summarized in Fig. 9, and technical characteristics and safety profiles of the new PFA catheter are described in subsequent sections. Varipulse is the only PFA system among the aforementioned devices that incorporates real-time force sensing during the ablation procedure. This suggests its potential advantages in a further optimization of the safety of the procedure.

## 5. Research Status of Novel PFA Ablation Catheters

### 5.1 Balloon-in-Basket PFA

The Volt system (Abbott) exemplifies the balloon-type PFA philosophy, integrating real-time three-dimensional (3D) electroanatomical mapping by the EnSite X EP System to achieve precise lesion creation. A prospective premarketing trial involving 32 patients revealed, an acute procedural success rate of 99.2% of this system with no major AEs [69]. Generally, balloon inflation mechanically removes the residual blood between electrodes and myocardium, theoretically reducing the hemolysis risk. However, current evidence remains limited to pulmonary vein isolation, with no data addressing the safety profile of the system during mitral/tricuspid isthmus ablation. This critical knowledge gap is probably being addressed in the ongoing VOLT-AF IDE clinical trial (NCT06223789), in which 435 patients were enrolled to comprehensively evaluate the system’s safety and efficacy among broader ablation targets.

### 5.2 Focal Linear PFA Ablation Catheter 

Next-generation focal ablation catheters, using a point-by-point ablation strategy, demonstrate improved safety profiles due to the ameliorated tissue contact. The CENTAURI system (Galvanize Therapeutics) currently represents the only clinically available single-tip linear catheter. A clinical study demonstrates promising outcomes, with 100% acute procedural success in complex atrial tachycardia cases and 73% freedom from atrial tachyarrhythmias at 6-month follow-up (82% for paroxysmal AF, 68% for persistent AF) [70]. As regards energy-specific complications, analysis of two studies involving 32 isthmus ablation procedures (15 anterior mitral lines, 2 lateral MI ablations, and 15 CTI ablations) revealed only one case of nitroglycerin-responsive right coronary artery spasm [71, 72]. Despite these preliminary results suggest favorable safety characteristics, a larger-scale investigation is needed to comprehensively evaluate energy-specific adverse events associated with focal PFA catheter because of the limitations posed by the small cohort size in current prospective studies.

### 5.3 PFA/RFA Catheter

The Sphere-9 catheter (Medtronic) is the first clinically available dual-energy system, features a unique catheter tip design consisting of a 9 mm diameter expandable NiTi-based lattice electrode with a spherical surface containing nine microelectrodes (each 0.7 mm in diameter). This lattice head end design allows for a larger single ablation area than conventional linear catheters.

A recent randomized trial demonstrated the noninferiority of Sphere-9 to RFA [73]. Adverse event analysis revealed transient phrenic nerve injury (0.9%) and bradycardia in the PFA group, versus hematuria (0.5%) in the radiofrequency group. However, the unique pulsed-field energy waveform of the Sphere-9 catheter, combined with its catheter design, increases the anisotropy of its emitted electric field, potentially contributing to coronary spasm mediated by atypical ablation sites. Del Monte A *et al*. [74] report that the Sphere-9 catheter induces progressive ST-segment increase in the inferior leads during the creation of a posterior MI line positioned immediately below the mitral annulus and subsequent coronary angiography reveals the subocclusion of the distal left circumflex coronary artery at a site corresponding to the prior pulsed-field application. The absence of routine pre-procedural nitroglycerin administration in this case may have increased the risk of coronary spasm, this observation emphasizes the need to recognize catheter-mediated coronary spasm occurring in atypical sites.

The ThermoCool SmartTouch Surroundflow-Dual-Energy catheter (Biosense Webster) uses advanced contact force monitoring and real-time 3D mapping abilities to quantitatively predict lesion depth with high accuracy in a preclinical model. This catheter represents a shift in ablation technology as the integrated PFA/RFA catheter system is able to perform real-time energy impact assessment through quantitative parameter analysis. This technological advancement enables precise control the distribution of PEF, potentially minimizing collateral tissue effects. Preliminary evidence from the SmartfIRE study demonstrated favorable safety profiles, with no coronary spasm events reported among 12 patients who underwent CTI ablation (n = 6 PFA-only, n = 6 RF-only) [75]. The unique abilities of the system, including quantitative lesion assessment through exponential modeling, alternative energy source availability, and stable tissue contact, may significantly reduce energy-specific AEs by optimizing the delivery parameters of PEF and minimizing unnecessary energy exposure.

### 5.4 Spherical Multielectrode Array PFA

Most of the first-generation PFA systems used in clinical practice lack the detection of real-time pressure, relying instead on fluoroscopic guidance combined with electroanatomical mapping for catheter positioning. This approach risks causing suboptimal tissue apposition, potentially resulting in unnecessary energy delivery through incorrectly positioned electrodes. The new map-and-ablate spherical array PFA catheter (Kardium Inc.) is ameliorated by protocols of contact force-guided selective activation protocols. This system integrates 122 gold-plated electrodes among 16 articulating ribs, synchronized with a proprietary Globe Pulsed Field System to enable the real-time assessment of tissue adherence assessment and selective electrode activation. A premarketing clinical study reveals an acceptable safety profile of the catheter demonstrated acceptable safety profile, as 11 patients who underwent extended ablation protocols (pulmonary vein isolation plus posterior wall and mitral isthmus ablation), did not show any instances of coronary spasm [76]. Our hypothesize that catheters incorporating this design may mitigate hemolysis by minimizing unnecessary discharges in the atrial blood pool. Such targeted energy delivery can reduce erythrocyte damage, though empirical validation through larger-scale studies should be performed.

### 5.5 Nanosecond PFA Technique

Nanosecond pulsed-field ablation (nsPFA) represents a transformative design in parameter optimization, distinct from conventional millisecond and microsecond PFA in its mechanism of action [77, 78, 79, 80]. This modality induces targeted apoptosis through intracellular effect while avoiding thermal effects and muscle contractions [78]. The underlying mechanism involves high-frequency nanosecond pulses causing sustained membrane potential distortion, preventing the restoration of Ca^2+^ concentration restoration during interstimulus intervals. This leads to the inhibition of voltage-gated sodium/calcium channel and subsequent nanoelectroporation of sarcolemmal and sarcoplasmic reticulum membranes [81, 82, 83, 84]. Although no clinical experiences in the use of nsPFA for cardiac ablation are available, preclinical studies demonstrate exceptional systemic tolerance. The CellFX Percutaneous Electrode System achieves therapeutic levels three times above clinical thresholds in porcine liver, kidney, and skeletal muscle models, with no significant alterations in urinalysis or serum chemistry [85]. The development of the CellFX Nano-PFA 360 Catheter Endocardial Ablation System (Pulse Biosciences) marks a milestone in nsPFA technology, with ongoing first-in-human trials (NCT06696170) evaluating the safety and efficacy in patients with paroxysmal AF.

## 6. Conclusions

PFA carries distinct energy-specific complications. Although standardized management protocols for these AEs remain undefined, the amelioration of a comprehensive strategy that integrates preoperative patient assessment, intraoperative precautions, and controlled energy delivery significantly mitigates serious risks. Next-generation PFA catheters featuring enhanced tip designs, optimized discharge parameters, and advanced ablation modalities offer substantial potential for a further reduction of AE incidence. Procedural safety may be established by detailed perioperative protocols specifically tailored to energy-specific AEs, requiring synergistic integration with evolving PFA systems.
